# Microclimate and Larval Habitat Density Predict Adult *Aedes albopictus* Abundance in Urban Areas

**DOI:** 10.4269/ajtmh.19-0220

**Published:** 2019-06-10

**Authors:** Michelle V. Evans, Carl W. Hintz, Lindsey Jones, Justine Shiau, Nicole Solano, John M. Drake, Courtney C. Murdock

**Affiliations:** 1 Odum School of Ecology, University of Georgia, Athens, Georgia;; 2 Center for Ecology of Infectious Diseases, University of Georgia, Athens, Georgia;; 3 Department of Applied Ecology, North Carolina State University, Raleigh, North Carolina;; 4 Department of Biology, Albany State University, Albany, Georgia;; 5 Department of Infectious Disease, University of Georgia, Athens, Georgia

## Abstract

The Asian tiger mosquito, *Aedes albopictus*, transmits several arboviruses of public health importance, including chikungunya and dengue. Since its introduction to the United States in 1985, the species has invaded more than 40 states, including temperate areas not previously at risk of *Aedes*-transmitted arboviruses. Mathematical models incorporate climatic variables in predictions of site-specific *Ae. albopictus* abundances to identify human populations at risk of disease. However, these models rely on coarse resolutions of environmental data that may not accurately represent the climatic profile experienced by mosquitoes in the field, particularly in climatically heterogeneous urban areas. In this study, we pair field surveys of larval and adult *Ae. albopictus* mosquitoes with site-specific microclimate data across a range of land use types to investigate the relationships between microclimate, density of larval habitat, and adult mosquito abundance and determine whether these relationships change across an urban gradient. We find no evidence for a difference in larval habitat density or adult abundance between rural, suburban, and urban land classes. Adult abundance increases with increasing larval habitat density, which itself is dependent on microclimate. Adult abundance is strongly explained by microclimate variables, demonstrating that theoretically derived, laboratory-parameterized relationships in ectotherm physiology apply to the field. Our results support the continued use of temperature-dependent models to predict *Ae. albopictus* abundance in urban areas.

## INTRODUCTION

The Asian tiger mosquito, *Aedes albopictus*, is an invasive mosquito that became established in the United States following its introduction in 1985.^1,2^ *Aedes albopictus* can transmit several pathogens of public health importance, including La Crosse,^3^ dengue,^4,5^ and chikungunya viruses.^6^ Unlike another vector of these diseases, *Aedes aegypti*, which originated in east Africa, *Ae. albopictus* originated from a temperate area of Asia and is able to survive in cooler climates than *Ae. aegypti.* Following initial establishment in Texas, *Ae. albopictus* has invaded more than 40 states,^7^ and models predict its range will expand as the climate warms.^8,9^ At present, established populations of *Ae. albopictus* are found in the United States as far north as Connecticut and New York,^10,11^ well outside the present range of *Ae. aegypti*. *Aedes albopictus* is implicated in transmission cycles of dengue and chikungunya in the Mediterranean region of Europe,^12,13^ which suggests that temperate regions of the United States may be similarly vulnerable.

Given the potential role of *Ae. albopictus* in disease transmission, it is important to understand what factors influence its abundance. *Ae. albopictus* is sensitive to variation in temperature because of temperature-dependent life history traits, such as development rates, fecundity, and survival.^14–16^ Climate or meteorological predictors are widely used in mechanistic models and statistical models.^17–22^ Models leverage these relationships to predict mosquito presence, population growth rates, and abundances based on temperature metrics derived from weather stations or remotely sensed datasets. However, urban landscapes are composed of a variety of land classes (e.g., residential, developed, and vegetated), which vary in their microclimates at fine spatial scales less than 1 × 1 km.^23–25^ This difference in microclimate can alter mosquito population growth rates,^26,27^ leading to variation in population abundances that may be missed by models that rely on coarser spatial data.

In addition, adult abundance may be determined by the abundance of larval habitat. *Ae. albopictus* is fairly nondiscriminate in its habitat use, and larvae are found in both natural and artificial containers.^11,28,29^ Several studies have found that adult abundance is positively related to the availability of larval habitats.^30,31^ This relationship is also the basis for larval source reduction techniques widely used in vector control.^32^ Urban microclimates can covary with the mosquito larval habitat density, which may differ in quality and quantity across urban land use.^26,30^ Thus, when studied independently, the relative roles of microclimate and larval habitat may be confounded.

Here, we combine field surveys of larval habitat and adult mosquito abundances with microclimate data to investigate how microclimate and the availability of larval habitat contribute to changes in adult *Ae. albopictus* abundance across an urban landscape. We aim to answer the following questions:

1. Does the density of larval habitat positive for *Ae. albopictus* change across urban land classes?
2. Does the abundance of *Ae. albopictus* adults change across urban land classes?
3. What is the relationship between microclimate and adult abundance?
4. What is the relationship between larval habitat and adult abundance?

By investigating these relationships, our results inform if and how predictive models should include microclimate variables and data on larval habitat from the field in their predictions of adult *Ae. albopictus* abundance. Furthermore, these results can help determine whether variation in land class alters the spatial distribution of *Ae. albopictus* and whether omitting this fine-scale variation may lead to bias in models.

## MATERIALS AND METHODS

**Figure 1.**
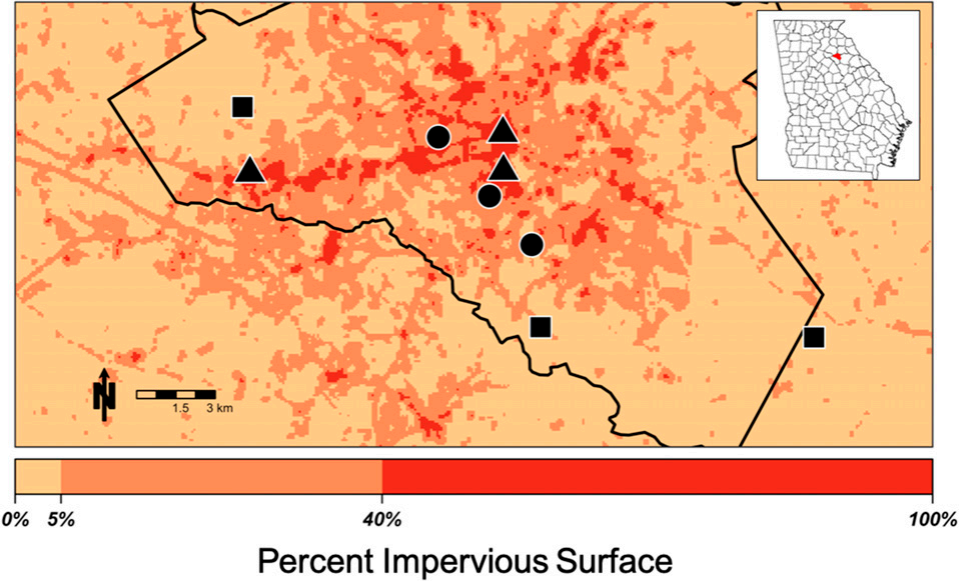
Map of sites in Athens, GA. Symbols represent land classes (square: rural, circle: suburban, and triangle: urban). Color shading represents the amount of impervious surface within the 210-m focal area of each pixel, as illustrated on the color bar on the bottom. Athens-Clarke County is outlined in black, and its location within the state is shown in the inset map of Georgia. This figure appears in color at www.ajtmh.org.

The study was conducted between June 2016 and December 2017 in Athens-Clarke County, GA. Athens-Clarke County is an urbanized area in a matrix of rural forested and agricultural land, representing a wide range of land classes. Following previous work,^33^ we used an impervious surface map (National Land Cover Database 2011) to select three replicate 30 × 30-m sites each of low (0–5%), intermediate (6–40%), and high (41–100%) impervious surface (Figure 1). Percent impervious surface, an accurate predictor of land surface temperature,^34^ was chosen to ensure the sites exhibited the full range of microclimates present in the city.

### Aquatic immature surveys.

At each site, we conducted surveys of the aquatic immature stages (larvae and pupae) biweekly from June to December 2016 and April to December 2017 to measure the density of positive larval habitats (e.g., number of larval habitats positive for *Ae. albopictus* larvae per 100 m radius site). Each site was sampled within 1 day, and the full sampling period of surveys took place over several days, with a sampling period referring to the week in which surveys were conducted. Study areas were defined as a 100-m radius surrounding the center of the focal 30 × 30-m site. Each study area was inspected for the presence of standing water (i.e., puddles, ponds, and artificial containers). Each body of water was assessed for the presence of immature mosquitoes visually and using dipping methods. If immature mosquitoes were present, samples were collected from that habitat. Because adult mosquitoes were sampled concurrently with larval habitat sampling, destructive sampling could bias the adult catch rate. For this reason, we collected measures of the presence or absence of *Ae. albopictus* per habitat by sampling a subset of the immature mosquitoes per habitat (ranging from 5 to 27 individuals per habitat). Immature mosquitoes were kept separated by habitat and returned to the laboratory, where they were placed in 50–100 mL deionized water in 8 oz. glass jars (Ball) and provided fish food (Hikari Cichlid Gold Mini Pellet) ad libitum to ensure high emergence rates. Larvae and pupae were reared to adulthood in an incubator (Percival Scientific) at 27 ± 0.5°C, 80 ± 5% relative humidity, and a 12:12-hour light–dark photocycle. Once mosquitoes emerged, they were immediately frozen at –20°C, separated by sex, and identified to species following Darsie and Ward.^35^ A habitat was determined “positive” for *Ae. albopictus* during a sampling period if a male or female *Ae. albopictus* mosquito was identified as emerging from the habitat.

### Adult trapping.

At each site, we trapped adults either biweekly (during the season of highest mosquito activity, June–November 2016 and June–November 2017) or monthly (December 2016–May 2017 and December 2017). During the period of highest mosquito activity, adult trapping was conducted within 1 week of immature surveys. One BG Sentinel-2 (Biogents, Regensburg, Germany) mosquito trap was deployed in the center of each 30 × 30-m site for two consecutive trap days per sampling period. Mosquito traps were baited with a BG-Lure cartridge (Biogents) and an octenol (1-octen-3-ol) lure inside the trap. Trapping was not conducted during precipitation events, and traps were placed under the cover of vegetation to increase catch rates. Because *Ae. albopictus* is a day-biting mosquito, the traps were run (with a battery-powered fan) from 06:00 hours to 22:00 hours. After each trap day, catch bags were collected and replaced with a new catch bag to reduce destruction of samples. Collected adults were taken back to the laboratory, frozen in a –20°C freezer, and separated by sex and identified to species following Darsie and Ward.^35^ Abundances for both trap days were combined to calculate the total abundance for that sampling period. The date of that sampling period is defined as the day on which the second catch bag was collected.

### Microclimate variables.

Within each 30 × 30-m site, we evenly distributed six data loggers (Radio Frequency Identification Temperature Track-It Logger; Monarch Instruments, Amherst, NH) to measure microclimate (e.g., site-specific climatic variables). Data loggers were placed in full shade under vegetation, approximately 0.9 m above the ground. The loggers recorded instantaneous temperature and relative humidity at 10-minute intervals. From the 10-minute data, we calculated daily minimum, mean, and maximum values for both temperature and relative humidity for each logger. These values were then averaged across all six loggers for each site. Intra-site variability among loggers was low, with an average of 0.402°C and 3.239% relative humidity variability within a site.

### Data analyses.

To determine if the density of positive *Ae. albopictus* larval habitat differed across land class, we used a generalized linear mixed model (GLMM) to test for the effect of land class on the density of positive larval habitats, including site as a random effect. The model included the week number of the study period as a basis spline (B-spline) function to account for seasonal differences in mosquito catch rates. The B-spline function allows a curve to be fit using maximum likelihood without prespecifying a function.^36^ A similar model was used to explore the effect of land class on *Ae. albopictus* adult abundance, again including site as a random effect and the week number of the study period as a B-spline function. Both models used a negative binomial distribution in which the variance increases quadratically with the mean:

$$\text{Var}\left(Y\right)=\mu +\left(\phi \times \mu^{2}\right),$$

where μ is the mean and ϕ is the dispersion parameter of the distribution.^37^ Models used a logarithmic link function. The statistical significance of land class effect was assessed by comparing fitted models to a null model that did not include land class as a predictor variable using a likelihood ratio test.

We used univariate GLMMs to investigate the effect of the microclimate variables on the density of positive larval habitat and adult abundance. We chose to use univariate analyses because of high correlation (*P* > 0.75) between variables of a similar measurement, such as between minimum and maximum temperature. Furthermore, a multivariate regression found no evidence of interactions between temperature and relative humidity for either the density of the positive larval habitat or the adult abundance (Supplemental Table 1). Temperature and relative humidity are interdependent, and our field-based study design is unable to control for this collinearity as could be performed in a laboratory setting. Microclimate variables were fit using a B-spline function to allow for nonlinear relationships, and site was included as a random effect. All models were fit with the quadratic variance form of the negative binomial distribution described previously and a logarithmic link function. We averaged each microclimate variable over the 7 days before surveying to account for the fact that captured mosquitoes likely developed and emerged within that time period. We explored using two different lag widths, 7 and 14 days, in the models. Resulting models did not differ significantly, and so, a lag of 7 days was used. This agrees with prior work in the same system that found mosquito development rates to range from 7 to 10 days during periods of high mosquito activity.^27^ This resulted in the following variables: mean weekly temperature and relative humidity, minimum weekly temperature and relative humidity, and maximum weekly temperature. Maximum weekly relative humidity was excluded from the analysis because 226 of 234 trap periods had a maximum value of 100% relative humidity. We included the day of mean relative humidity value in models of adult abundance to control for mosquito activity on that trap day. We assessed the statistical significance of each microclimate variable by comparing fitted models to a null model that did not include the variable as a predictor variable using a likelihood ratio test.

We also tested for the effect of the density of positive *Ae. albopictus* larval habitat (the number of larval habitats that had *Ae. albopictus* larvae present per site) on adult abundance within a site and sampling period. A GLMM was fit including the density of positive habitats as a predictor variable and site as a random effect. We fit the model with the same negative binomial distribution and logarithmic link function as described previously.

All GLMMs were fit using the *glmmTMB* package in R version 3.5.2.^38,39^ Scaled residuals of the models were inspected for overdispersion and uniformity using the *DHARMa* package.^40^ Code and data to reproduce analyses are deposited on the figshare repository (doi:10.6084/m9.figshare.7869353).

## RESULTS

A total of 1,107 adult female *Ae. albopictus* mosquitoes were sampled from May 2016 to December 2017, encompassing 468 trap nights over two seasons of mosquito activity. This resulted in 26 adult sampling events for each of the nine sites, or 78 sampling events per land class. We sampled each site for larval habitat a total of 21 times and found 217 habitats positive for *Ae. albopictus* across all nine sites. Whereas 92.57% of adult mosquitoes sampled were *Ae. albopictus*, the second and third most common species were *Culex quinquefasciatus* (5.45%) and *Aedes triseriatus* (0.92%), respectively. Similarly, *Cx. quinquefasciatus* and *Ae. triseriatus* were found in 61 and 36 larval habitats, respectively.

### Land class and season.

**Figure 2.**
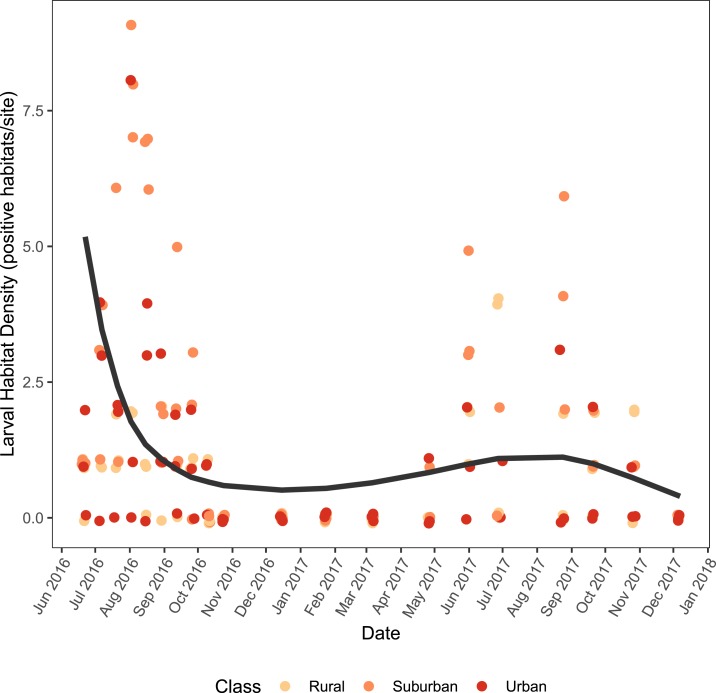
Positive larval habitat density across land class and time. The lines represent the fitted model averaged across all sites. Raw data are represented by the points and randomly jittered to improve visibility. This figure appears in color at www.ajtmh.org.

The density of larval habitats positive for *Ae. albopictus* was highly seasonal, peaking in June–August of both years (Figure 2). The best fitting B-spline used a three-degree polynomial, and the effect of sampling week was significant (χ^2^ = 37.023, df = 3, *P*-value < 0.001). Although suburban sites tended to have a higher density of positive larval habitat than rural and urban sites, this difference was not significant. A null model without land class as a predictor variable was not significantly different from the full model (χ^2^ = 4.34, df = 2, *P*-value = 0.110) and predictive performance was similar ($R_{\text{NULL}}^{2}$ = 0.503, $R_{\text{FULL}}^{2}$ = 0.483).

**Figure 3.**
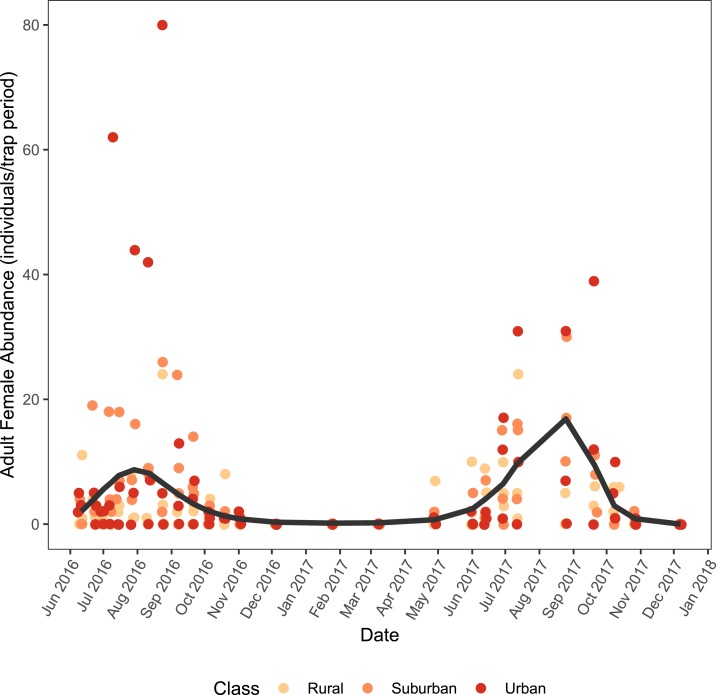
Adult female *Aedes albopictus* abundance across time. The lines represent the fitted model averaged across all sites. Raw data are represented by the points and randomly jittered to improve visibility. This figure appears in color at www.ajtmh.org.

We found evidence of very strong seasonality in adult *Ae. albopictus* density across all sites, with densities peaking in July and August of both years (Figure 3). There was a significant effect of the sample week on adult density (χ^2^ = 112.050, df = 4, *P*-value < 0.001), and the best fitting B-spline had a four-degree polynomial. There was no evidence for a difference in adult *Ae. albopictus* density across land class. The null model without land class as a predictor variable was not significantly different from the full model (χ^2^ = 0.602, df = 2, *P*-value = 0.740) and performed similarly ($R_{\text{NULL}}^{2}$ = 0.813, $R_{\text{FULL}}^{2}$ = 0.813).

### Microclimate and larval habitat density.

**Figure 4.**
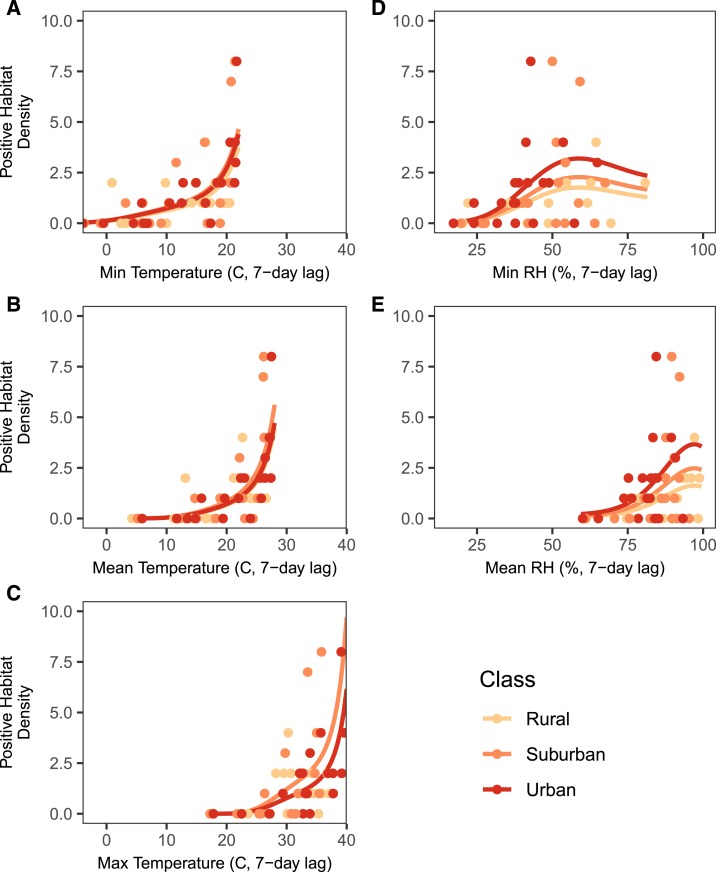
Functional relationship between microclimate variables and the density of the positive *Aedes albopictus* habitat for a representative site of each land class. Functional relationships were the same across all land classes, and the larval habitat density did not differ across land class. The daily minimum, mean, and maximum temperatures are on the left side (**A**–**C**), and the daily minimum and mean relative humidity are on the right side (**D** and **E**). The lines represent fitted regression lines, and raw data are represented by the circles. Because the maximum relative humidity did not vary, no regression line was fitted. This figure appears in color at www.ajtmh.org.

Univariate analyses revealed a significant, nonlinear relationship between all microclimate variables and the density of positive *Ae. albopictus* habitat (Table 1, Figure 4). The density of positive larval habitat increased with increasing minimum, mean, and maximum temperatures (Figure 4). The larval habitat increased with increasing minimum relative humidity until approximately 60% relative humidity, after which increasing relative humidity was associated with fewer larval habitats (Figure 4). The relationship between mean relative humidity and the density of larval habitats was similarly unimodal, although its optimum neared 100% relative humidity. Importantly, the functional forms of these relationships differ from those between the microclimate variables and adult abundance. This difference suggests that the effects of microclimate on oviposition behavior and habitat availability differ from the effect of microclimate on mosquito emergence and adult longevity.

**Table 1:** Results of likelihood ratio tests comparing a null model to a univariate generalized linear mixed model containing microclimate variables to predict the density of the *Aedes albopictus*–positive larval habitat

Variable	df	χ^2^	*P*-value	Conditional *R*^2^
Minimum temperature	3	93.516	< 0.0001	0.764
Mean temperature	3	92.189	< 0.0001	0.830
Maximum temperature	3	61.480	< 0.0001	0.855
Minimum relative humidity	3	52.522	< 0.0001	0.695
Mean relative humidity	3	24.776	< 0.0001	0.506

All microclimate variables were strong predictors of larval habitat density. The generalized linear mixed model was calculated across nine sites (random effect) and within-site (*n* = 21). We calculated conditional *R*^2^ following Schielzeth and Nakagawa.^60^

### Microclimate and adult abundance.

**Figure 5.**
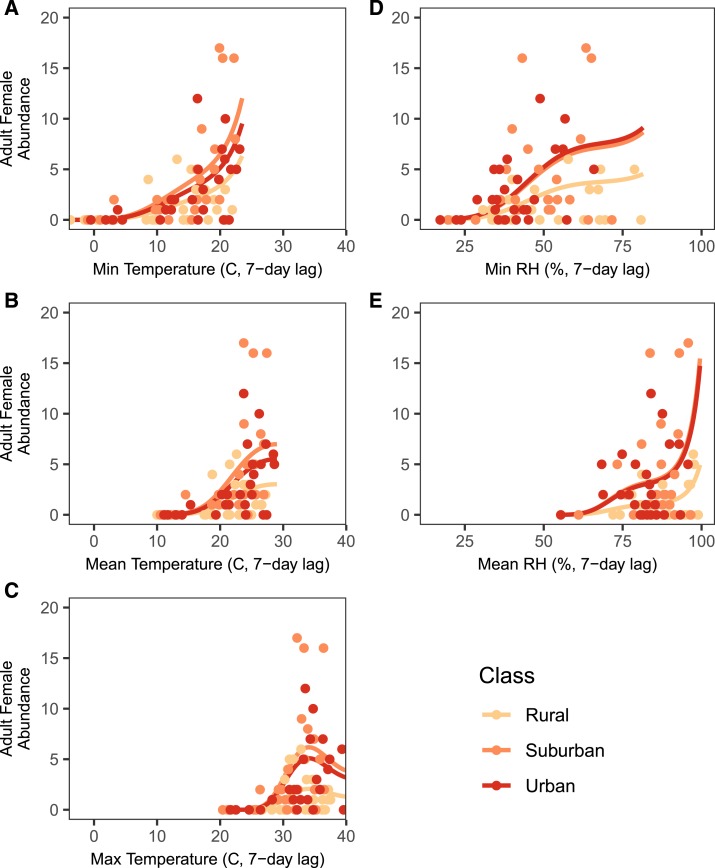
Functional relationship between microclimate variables and adult female abundance for a representative site of each land class. Functional relationships were the same across all land classes, and the female adult abundance did not differ across land class. The daily minimum, mean, and maximum temperatures are the on the left side (**A**–**C**), and the daily minimum and mean relative humidity are on the right side (**D** and **E**). The lines represent fitted regression lines, and raw data are represented by the circles. Because maximum relative humidity did not vary, no regression line was fitted. The suburban and urban fitted curves for minimum relative humidity are visually indistinguishable, and so, the suburban curve has been shifted downward for visibility. This figure appears in color at www.ajtmh.org.

Univariate analyses revealed that all five microclimate variables significantly influenced adult abundances (Table 2). The relationships between microclimate variables and adult abundance were nonlinear for all variables (Figure 5). Mean and minimum relative humidity had a third-order B-spline fit that increased exponentially as relative humidity approached 100% (Figure 5). All three temperature variables also had a third-order B-spline fit, evidence of a nonlinear relationship. The minimum daily temperature was similar to relative humidity in that it was an increasing function across the range measured in this experiment (−3.75 to 23.10°C). The functional relationships between mean and maximum temperature and adult abundance were unimodal, decreasing after an optimal temperature threshold was reached (Figure 5).

**Table 2:** Results of likelihood ratio tests comparing a null model to a univariate generalized linear mixed model containing microclimate variables to predict adult female abundance

Variable	df	χ^2^	*P*-value	Conditional *R*^2^
Minimum temperature	3	104.27	< 0.001	0.835
Mean temperature	3	110.94	< 0.0001	0.847
Maximum temperature	3	96.50	< 0.0001	0.910
Minimum relative humidity	3	49.79	< 0.0001	0.608
Mean relative humidity	3	16.257	0.001	0.530

All microclimate variables were strong predictors of adult abundance. Generalized linear mixed model was calculated across nine sites (random effect) and within-site (*n* = 26). We calculated conditional *R*^2^ following Schielzeth and Nakagawa.^60^

### Larval habitat density and adult abundance.

We detected a significant positive relationship between larval habitat density and adult abundance at a site (χ^2^ = 17.788, df = 1, *P* < 0.0001), although the effect size was highly dependent on site. Site-level random effects ranged from –0.975 to 1.280, compared with a regression coefficient of 0.364, suggesting that unmeasured covariates at the site level are also contributing substantially to adult abundances. This is further supported by the relatively low model fit (*R*^2^ = 0.39) compared with the univariate models of microclimate variables described previously.

## DISCUSSION

Spatial predictions of mosquito abundances often rely on temperature-dependent mechanistic models derived from mosquitoes’ thermal performance curves.^17–19^ However, availability of larval habitat can also be a strong determinant of adult mosquito abundances, and few models include these in their predictions (but see ref. 19). We found that although both climate and larval habitat influenced adult mosquito abundance, climate was a stronger predictor of adult abundance and the functional relationship between microclimate and adult abundance matches predictions based on theories of ectotherm physiology.^41^ Furthermore, neither adult abundance nor the density of *Ae. albopictus*–positive larval habitat varied by urban land class, suggesting that, at least for smaller, residential cities, this variation is not significant, and models do not need to differentiate across land class in urban areas.

Although we failed to detect a difference in adult abundance across land class, other studies have found mosquito abundances to differ across an urban gradient. Multiple studies that have defined urban gradients according to variation in vegetation density,^42,43^ impervious surface,^44^ or land cover classifications^45–48^ have found mosquito abundances to vary across these gradients. Li et al.^26^ focused specifically on *Ae. albopictus* and found adult abundance to increase with increasing urbanization, driven by an increase in larval habitat density. Our study, however, found no evidence for a difference in positive larval habitat density across land classes, which may explain why we failed to detect a difference in adult abundance. A semi-field experiment conducted at the same study sites as this experiment estimated lower *Ae. albopictus* per capita growth rates on urban sites than rural and suburban sites, driven by lower larval survival rates and smaller wing lengths of emerged adults (a predictor of fecundity) on urban sites.^17^ Taken together, these findings suggest that per capita growth rates may not scale-up to site-level population abundances. Other factors, such as the quantity and quality of larval habitat or the availability of hosts for blood feeding,^49^ may further mediate the relationship between container-level growth rates and site-level abundances.

Temperature and relative humidity are likely the key variables driving seasonal trends in mosquito density, as they were important predictors of both the density of larval habitats positive for *Ae. albopictus* larvae and adult *Ae. albopictus* abundance. Although many studies have observed seasonality in larval habitat abundance,^50,51^ few have directly paired these data with climate variables. The density of larval habitat had an exponentially increasing relationship with temperature, and indeed, larval habitat was most abundant during the summer sampling periods. Hotter temperatures can increase mosquito biting rates and shorten gonotrophic cycles,^14^ potentially leading to higher oviposition rates and a higher density of larval habitats. Relative humidity, in comparison, had a unimodal relationship with larval habitat, with the number of larval habitats decreasing at high minimum and mean relative humidity. Very few studies have investigated the effects of relative humidity on larval mosquito dynamics. However, Murdock et al.^27^ found that increases in relative humidity reduced larval survival through a suggested decrease in the surface tension of aquatic environments. Another explanation is that high relative humidity is associated with strong rainfall events,^52^ which can lower the density of the positive larval habitat through flushing events and oviposition avoidance.^53^ Interestingly, these functional relationships (Figure 4) differed qualitatively from those between climate and adult abundance (Figure 5), suggesting the effects of temperature and relative humidity on mosquito populations may differ across life stages (ovipositing and hatching versus emergence and adult survival).

Temperature and relative humidity were also key predictors of adult abundance. The nonlinear functions used in the temperature models match the unimodal functional form between ectotherm growth and temperature expected from physiological theory and empirical work in mosquito systems.^17,41^ *Aedes albopictus* abundance was zero at mean temperatures below 10°C and increased to a peak temperature around 25°C. This agrees with other studies in urban areas that found the minimum threshold for adult activity to be 10°C and laboratory predictions of the optimum temperature of 25°C.^54,55^ In the case of the daily maximum temperature, temperatures during our study period exceeded the optimal temperature for *Ae. albopictus* and adult abundance decreased at high temperatures, creating a hump-shaped curve. These field findings match general expectations of thermal performance curves derived from laboratory experiments, suggesting that empirically derived thermal performance curves are applicable to mosquito populations in field settings. Adult abundance also increased with increasing levels of relative humidity. Another study observed adult *Ae. albopictus* mortality rates to decrease with increasing humidity in the field.^56^ This relationship between adult mortality and humidity may drive the positive relationship between relative humidity and adult abundance in our study. We found a positive, although weak, relationship between the density of positive larval habitat and adult mosquito densities. This is in agreement with other studies that have found larval habitat to be predictive of adult densities.^26,31^ In addition to providing more space and resources for immature mosquitoes, high densities of larval habitat can also reduce the time spent searching for oviposition sites, shortening gonotrophic cycles and increasing population growth rates.^57^ The overall performance of the model including larval habitat was lower than the one based solely on microclimate. This implies that although adult abundance and larval habitat are correlated, microclimate alone may more accurately predict mosquito abundances.

By spanning 2 years, we replicated seasonality, but only across a limited number of sites. Although we classified sites into urban land classes based on determinants of microclimate, namely, impervious surface, unmeasured site-level characteristics were an important driver of *Ae. albopictus* abundance. For example, one urban site produced more than 2-fold the number mosquitoes of any other urban site. This site received daily irrigation throughout the summer months, perhaps contributing to high *Ae. albopictus* abundances, as has been found in *Culex* spp.^58^ The types of artificial containers can differ across socioeconomic levels in urban areas.^42^ The type of larval habitat in our study varied widely across sites, from natural bodies such as ponds and treeholes to artificial containers such as flower pots and tires, but there was no pattern across land class (Supplemental Table 2). Suburban and urban sites in particular had wide variation in habitat types, and the inclusion of social variables such as parcel value or income in our classification could lead to higher uniformity in land classifications.

We found that adult abundance was well predicted by microclimate variables and that the functional relationship between temperature and adult abundance matched that proposed by theory and empirical studies. This study contributes to a small number of studies exploring predictors of *Ae. albopictus* abundance in cities.^26,42^ Unlike past studies, we found no evidence for an effect of urban land class on *Ae. albopictus* abundances, suggesting that city-scale predictive models may not need to explicitly incorporate differences across land classes. However, Athens, GA, is a small city, with an average impervious surface of 10% and a population of 127,064, and these results may not apply to larger cities with wider variation in land class, which can differ in temperature by more than 5°C.^59^ Future work could expand field studies to additional cities to test the generalizability of these findings and identify contexts (e.g., tropical versus temperate cities and small versus large cities) in which these results differ. By pairing mosquito surveys with the collection of microclimate data, our findings support the continued use of temperature-dependent mechanistic models in the spatial prediction of mosquito abundances and mosquito-borne disease risk.

## Supplemental materials

Supplemental tables and figures
