# Supplementary material for: Microclimate and Larval Habitat Density Predict Adult Aedes albopictus Abundance in Urban Areas
Source: Am J Trop Med Hyg. 2019 Jun 10;101(2):362–70. doi: 10.4269/ajtmh.19-0220 (PMC6685558; doi:10.4269/ajtmh.19-0220)
Supplement: Supplementary file 1 [file tpmd190220.SD1.pdf]

The following are supplemental materials and will be published online only

SUPPLEMENTAL TABLES

**Model**	**Variables**	**df**	χ^2	**p-value**	**Δ AIC**
Larval Habitat Density	Mean Temp. * Mean RH	9	16.550	0.056	1.45
Adult Abundance	Mean Temp. * Mean RH	3	6.368	0.703	11.47

Table S1. Results of likelihood ratio tests comparing models with interactions to main effect GLMMs containing microclimate variables to predict the density of *Ae. albopictus*-positive larval habitat or adult *Ae. albopictus* abundance. There was no evidence for an interaction between temperature and relative humidity for either model, as demonstrated by the χ^2 value and comparison of AIC. GLMM was calculated across 9 sites (random effect).

	**Artificial Container**	**Ground Pool**	**Pond**	**Rockpool**	**Treehole**
**Rural**	46/201	4/12	1/6	0/0	0/5
**Suburban**	111/378	0/5	0/20	1/2	3/32
**Urban**	28/129	0/22	1/35	0/0	17/38

Table S2. Distribution of container types across land classes (positive/total). Most container types were found across all land classes, with the exception of rockpools.
